# Mountain Building Triggered Late Cretaceous North American Megaherbivore Dinosaur Radiation

**DOI:** 10.1371/journal.pone.0042135

**Published:** 2012-08-02

**Authors:** Terry A. Gates, Albert Prieto-Márquez, Lindsay E. Zanno

**Affiliations:** 1 Ohio University College of Osteopathic Medicine, Athens, Ohio, United States of America; 2 North Carolina State University, Raleigh, North Carolina, United States of America; 3 Bayerische Staatssammlung für Paläontologie und Geologie, Munich, Germany; 4 Nature Research Center, North Carolina Museum of Natural Sciences, Raleigh, North Carolina, United States of America; University of Pennsylvania, United States of America

## Abstract

Prior studies of Mesozoic biodiversity document a diversity peak for dinosaur species in the Campanian stage of the Late Cretaceous, yet have failed to provide explicit causal mechanisms. We provide evidence that a marked increase in North American dinosaur biodiversity can be attributed to dynamic orogenic episodes within the Western Interior Basin (WIB). Detailed fossil occurrences document an association between the shift from Sevier-style, latitudinally arrayed basins to smaller Laramide-style, longitudinally arrayed basins and a well substantiated decreased geographic range/increased taxonomic diversity of megaherbivorous dinosaur species. Dispersal-vicariance analysis demonstrates that the nearly identical biogeographic histories of the megaherbivorous dinosaur clades Ceratopsidae and Hadrosauridae are attributable to rapid diversification events within restricted basins and that isolation events are contemporaneous with known tectonic activity in the region. SymmeTREE analysis indicates that megaherbivorous dinosaur clades exhibited significant variation in diversification rates throughout the Late Cretaceous. Phylogenetic divergence estimates of fossil clades offer a new lower boundary on Laramide surficial deformation that precedes estimates based on sedimentological data alone.

## Introduction

Studies of dinosaur diversification and distribution are generally conducted from a global perspective [1]–[4] because poor stratigraphic resolution within individual continents has thus far prevented finer-scaled paleobiogeographical analyses. Yet, understanding the dynamics of dinosaurian evolution at the intracontinental scale is required to identify correlative factors that may have driven lineage diversification at more inclusive levels.

Key studies [5], [6] have established the Late Cretaceous Western Interior Basin (WIB) of North America as the most detailed chronostratigraphic framework available worldwide for fine-scale intracontinental studies of dinosaur biostratigraphy and biogeography. To date, little work has been conducted investigating fine-scale patterns of Laramidian dinosaur biodiversity at the subclade level. Gates et al. [7] found that late Campanian (76–74 Ma) North American dinosaur species exhibited a more restricted geographic distribution than expected for vertebrates of large-body size [8]. This result contrasts with the more cosmopolitan biogeographic patterns of Maastrichtian dinosaurs [9]. Similarly, global dinosaur diversity has been estimated as higher during the Campanian relative to the Maastrichtian [10]–[14], yet little substantial evidence has been presented documenting potential causal factors for changing biodiversity patterns.

Among Late Cretaceous dinosaurs, the megaherbivorous hadrosaurid (duck-billed) and ceratopsid (horned) dinosaur clades provide exemplary case studies for examining macroevolutionary patterns because these clades are the most species-rich and possess refined phylogenetic relationships unparalleled in stratigraphic and geographic controls [15]–[17].

Here, we test for factors associated with the Campanian biodiversity surge within the WIB of North America–specifically the effects of mountain uplift on megaherbivorous dinosaur net diversification–within the most detailed biostratigraphic and biogeographic framework yet compiled for megaherbivorous dinosaurs.

### Geological Context and Orogenic Speciation

**Figure 1:**
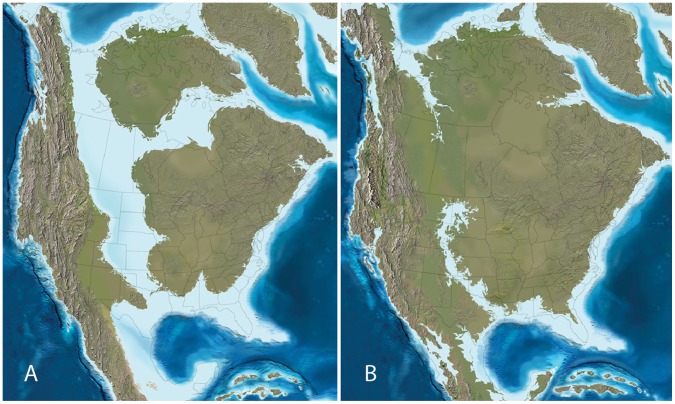
Paleogeographic maps of North America during the (A) late Campanian (∼75 Ma) and (B) late Maastrichtian (∼65 Ma). The Sevier Orogenic Belt is the major mountain building system in western North America during the late Campanian, but note that by the latest Maastrichtian the Laramide Orogeny creates uplift structures further to the east. Maps courtesy of Ron Blakey, Colorado Plateau Geosystems.

During much of the Late Cretaceous, an epeiric intercontinental seaway (KWIS) inundated the central portion of North America, splitting the landmass into two island continents, Appalachia to the east and Laramidia to the west. Laramidia was further bounded on the west by the Sevier Orogenic belt (Fig. 1A). The Cretaceous–Neogene Laramide orogeny produced the Rocky Mountains of western North America, which are major physiographic features dictating modern climatic regimes and biogeographic patterns in this region [18]. Uplift began when the subducting Farallon tectonic plate shifted from a deeper to a more shallow position. This shift ultimately moved tectonic stresses further into the continental interior [19]–[21], leading to the uplift of mountains east of the Sevier Orogenic Belt (Fig. 1B). During the Late Cretaceous, inception of Laramide tectonics altered the topography of the WIB from an extensive foreland basin into several smaller basins positioned east of the Sevier range and caused regression of the KWIS from its position in the center of North America at the close of the Campanian [22].

Varied techniques are utilized to date the onset of Laramide uplift. Isotopic ages from metamorphic or volcanic rocks provide a minimum time for the emplacement of uplifted structures [23], [24] and shifts in sedimentary basin drainage and subsidence allow relative dating of changes in drainage basin profile and source rock, which can be used to date emplacement of major topological alterations [5], [20], [22], [25]–[29]. However, geologic evidence for uplift may be delayed relative to its earliest phases as a result of weathering and eroding uplifted rock, shifting river directions, and depositing subsequent sediments. Organismal evolution offers an independent line of evidence for the emplacement of major topographical features, which may prove useful in refining the timing of events substantiated by the geologic record. Refinement in the timing of uplift may be possible when biological evidence is taken into account because species are known to respond rapidly to environmental disturbance, with documented genetic differences appearing on the scale of mere decades [30].

Numerous studies have documented a relationship between orogenic activity and speciation rate [31]–[37]. These studies range from demonstrating changes associated with topographic complexity, proximity to orogenic centers, and coincidence of mountain uplift and phylogenetic patterns. For example, Miller and Mao [32] proposed that diversity of Ordovician invertebrate genera increased near orogenic centers. Peters [38] demonstrated that the macroevolutionary history of marine animals is driven by the creation and cessation of sedimentary basins regulated by eustatic and tectonic controls. Also, Finarelli and Badgley [37] showed that Miocene rodents occupying the Great Plains diversified at a slower rate than those living in the tectonically active Basin and Range province.

Several additional studies have focused on using evolutionary data to address the timing of orogenic events more specifically. For instance, Antonelli et al. [39] used the diversification pattern of South American coffee plants to corroborate geologic evidence of multiple episodes of Andean uplift. Likewise, Che et al. [36] used frog phylogenetics and environmental tolerance to time initial uplift of the Himalayan Mountains.

## Materials and Methods

### Reconstruction of Ancestral Areas

Ancestral areas for the clades recovered in the ceratopsid [40] and hadrosaurid [4] phylogenies were inferred via dispersal-vicariance analysis (DIVA 1.1) [41] using the exact search according to the optimization algorithm of Ronquist [42]. DIVA is an event-based technique that integrates phylogenetic information with explicit models of the processes that shape the distribution of taxa [43]. The program assumes allopatric speciation due to vicariance as a null hypothesis; however, the method also considers dispersal and extinction as alternative processes influencing the resulting distribution of taxa. DIVA uses a model in which vicariance, sympatric speciation, dispersal, and extinction events are given different costs. These costs are inversely related to the likelihood of occurrence of these events [44]. Specifically, vicariance (speciation due to emergence of a dispersal barrier) and duplication (speciation within the same area) have a cost of zero, whereas dispersal and extinction events have a cost of one per each area unit added or deleted, respectively, from the distribution [42]. DIVA uses parsimony as optimality criterion and searches for the reconstruction that minimizes the number of dispersal-extinction events (or cost) required to explain the geographical distribution of terminal taxa [42]. This procedure is accomplished via optimization of a three-dimensional cost matrix, where the cost of an event depends on the combination of the distributions of the sister taxa descended from a common ancestor [42]. In order to allow for the possibility of widespread ancestors, the number of ancestral areas optimized for a particular node was left unrestricted. Node ages were derived from the literature. We considered the following discrete general areas in which these taxa have been recorded: Northern and Southern WIB of North America, Eastern North America, South America, Europe, and Asia.

### Diversification Rates

The same phylogenetic trees utilized in DIVA were used to test for statistically significant variations in diversification rates in saurolophine hadrosaurids and chasmosaurine ceratopsids using the program SymmeTREE version 1.0 [45]. This program implements topology-based techniques that allow for detection of significant variations in diversification among the lineages of a given phylogeny. It does so by comparing the observed topological distribution of the taxon diversity to the expectations according to an equal-rates model [45]. Furthermore, SymmeTREE incorporates the topological distribution of the diversity the taxa of the entire tree, as well as various tests that use information on the relative diversity of the internal nodes of the phylogeny [45].

## Results and Discussion

**Figure 2:**
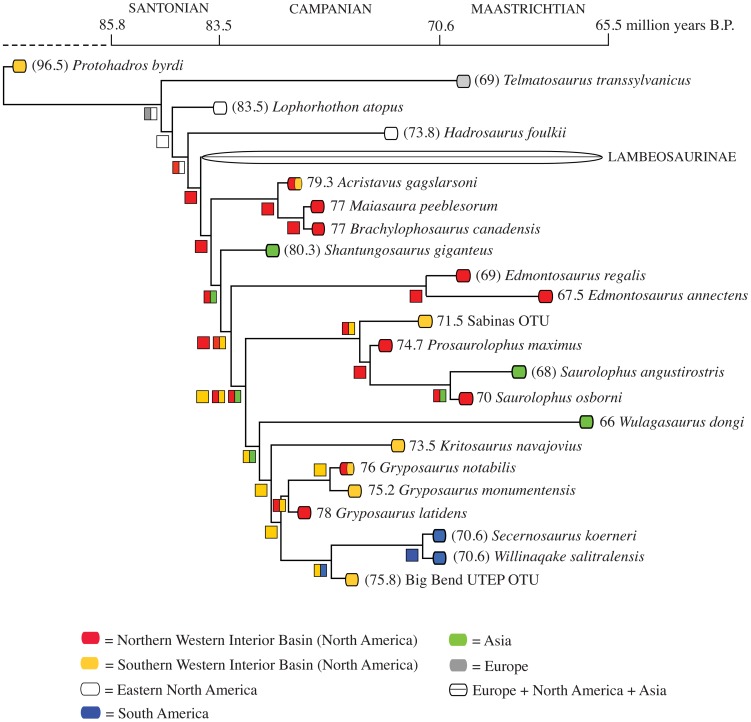
Saurolophine hadrosaurid phylogeny from Prieto-Márquez [**49**] with taxa time calibrated to known geologic occurrences. Numbers in brackets indicate the midpoint of a geologic stage that a taxon is known to occur if that species does not have more constrained stratigraphic ages. Symbols on phylogenetic branches designate the inheritance of geographic distribution based on results from the DIVA analyses.

**Figure 3:**
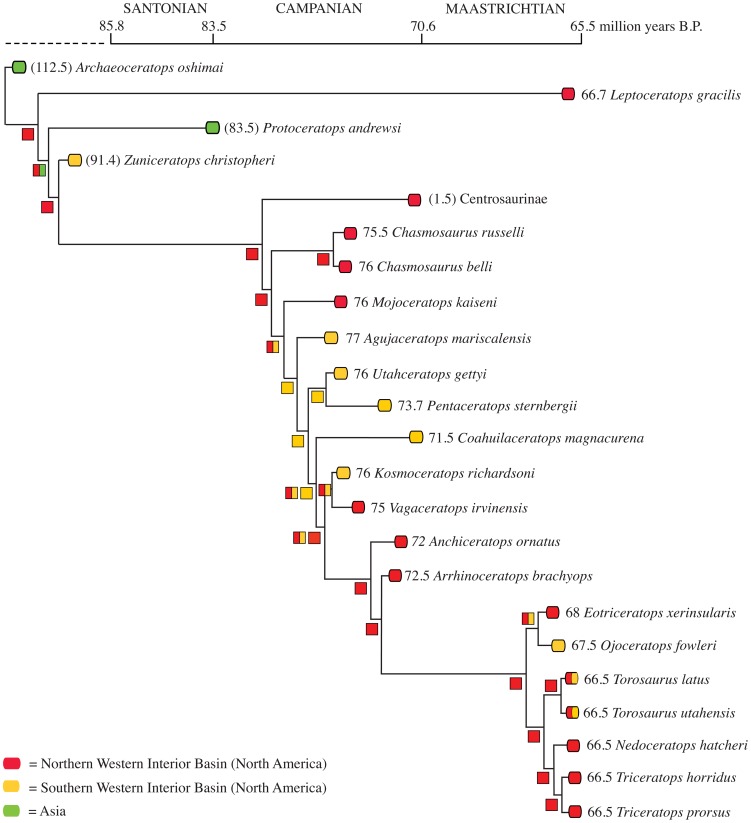
Chasmosaurine ceratopsid phylogeny from Sampson et al. [40] with taxa time calibrated to known geologic occurrences. Numbers in brackets indicate the midpoint of a geologic stage that a taxon is known to occur if that species does not have more constrained stratigraphic ages. Symbols on phylogenetic branches designate the inheritance of geographic distribution based on results from the DIVA analyses. Note that *Torosaurus* and *Triceratops* both appeared on the Sampson et al. [40] phylogeny used in this study; and that in light of recent work documenting these taxa as congeneric [68], they remain as distinct genera within the present figure in order to maintain the original data integrity of the Sampson et al. [40] study, but are considered and discussed here as solely *Triceratops*.

Both hadrosaurids and ceratopsids demonstrate segregated evolutionary centers between approximately 76.0–75.5 million years, with subsequent integration of northern and southern faunas later in the Campanian and early Maastrichtian. Time calibrated phylogenies of these two groups provide compelling evidence for a north-south isolation event at approximately 79–78 Ma (Figs. 2 and 3). Additionally, whole tree statistics from SymmeTREE analysis revealed that both saurolophines and chasmosaurines show significant variation in diversification rates (p = 4.1×10^−4^) throughout the Late Cretaceous (SymmeTREE Information S1). These results demonstrate dynamic rates of evolution in megaherbivores within the Campanian and Maastrichtian that are likely attributable to changing environmental factors.

### Hadrosaurid Campanian Biogeography

Our DIVA analyses posit the most recent common ancestor of saurolophine hadrosaurids as inhabiting the Northern WIB no later than the early Campanian (Fig. 2). The occurrence of taxa from the *Edmontosaurus*-*Gryposaurus* clade of saurolophines can be explained by either vicariance of a widespread ancestor or dispersal from the Northern WIB to the Southern WIB, no later than the early Campanian. The major speciation events of North American taxa within both the *Prosaurolophus* and *Gryposaurus* subclades are unambiguously inferred to have been the result of vicariance during Campanian times.

**Figure 4:**
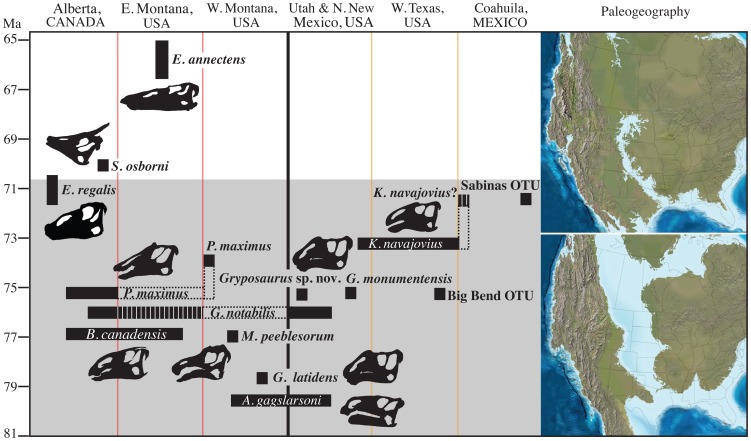
Saurolophine range distribution throughout the Western Interior Basin during the Campanian (lower grey area) and Maastrichtian (upper white area). To the right, paleogeographic maps of North America during the late Campanian (∼75 Ma) and late Maastrichtian (∼65 Ma). The sources for the geographic and stratigraphic position of the hadrosaurid species are as follows: *Acristavus gagslarsoni* [46], *Brachylophosaurus Canadensis* [81], *Edmontosaurus annectens* and *E. regalis* [82], *Gryposaurus latidens* [83], *G. monumentensis* [47], *G. notabilis* [15], *Gryposaurus* new species [84], *Kritosaurus navajovius* [85], [86], *Maiasaura peeblesorum* [81], *Prosaurolophus maximus* [15], [49], Sabinas OTU [4], [49], *Saurolophus osborni* [15], [87], and UTEP OTU [49], [88]. Maps courtesy of Ron Blakey, Colorado Plateau Geosystems.

The occurrence of *Acristavus gagslarsoni* in virtually coeval sediments (79.4, 79.3 Ma) from northwestern Montana and southern Utah [46] and *Gryposaurus notabilis* in both southern Alberta and southern Utah at approximately 76.5–76.0 Ma [17] demonstrates widespread distribution of saurolophine hadrosaurid dinosaurs throughout the early and middle Campanian (Fig. 4). At approximately 75.5 Ma, the geographic distribution of WIB hadrosaurids changes to one of localized species ranges. In southern Utah, a new species of *Gryposaurus*–*G. monumentensis–*appears in the fossil record [47], while at the same time in Alberta the species *Prosaurolophus maximus* makes its first appearance [15], [48] (Fig. 4). These taxa are members of geographically isolated sister clades (Fig. 2), suggesting they diverged from a common ancestor and represent separate evolutionary centers of diversification. No species of hadrosaurid belonging to the northern *Prosaurolophus* clade is known from the southern WIB until 71.5–71.0 Ma [49].

**Figure 5:**
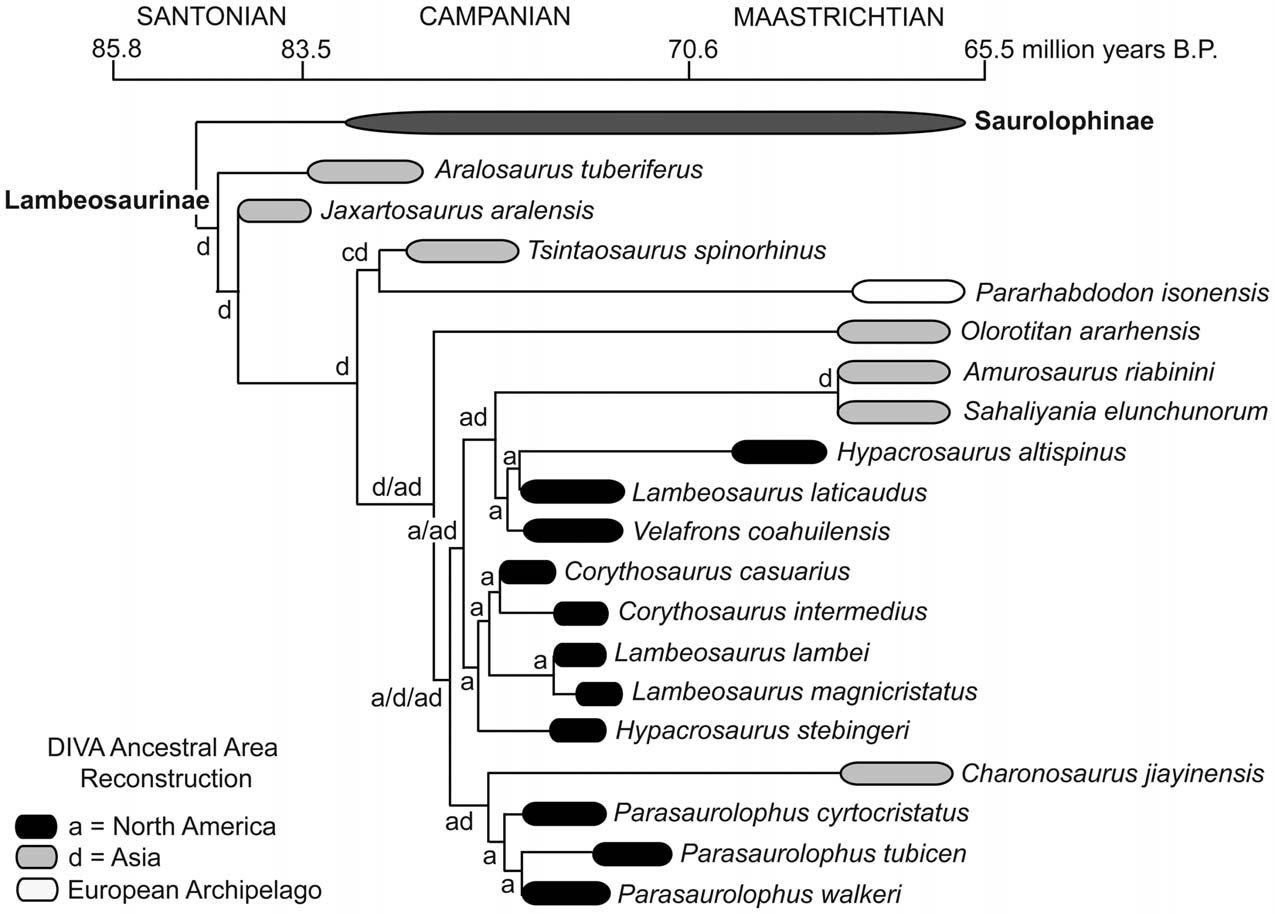
Time calibrated phylogeny of lambeosaurine hadrosaurids. Data from Prieto-Márquez et al. [52].

Specimens of the Lambeosaurinae clade of hadrosaurid dinosaurs are much rarer in sediments older than 76 Ma but a useful biogeographical pattern still is observable between 76–73 Ma. The tube-crested lambeosaurine *Parasaurolophus walkeri* makes a rare appearance in the Dinosaur Park Formation of Alberta around 76 Ma, which occupies the lower megaherbivore faunal zone with its crested cohort *Corythosaurus casuarius* [15], [48], [50]. These two lambeosaurines resolve in separate clades (Fig. 5). Other species closely related to *C. casuarius* are known from the northern WIB until the mid-Maastrichtian [15], [51], [52], after which lambeosaurine hadrosaurids apparently go extinct in North America. To date, the only lambeosaurines known from the southern portion of the WIB confidently ranging between 76–73 Ma are *Magnapaulia* [52], [53] and species of the genus *Parasaurolophus*. An unidentified species of this genus has been found within the Kaiparowits Formation dating from around 76 Ma [17]; *P. cyrtocristatus* is known from the Fruitland Formation of northern New Mexico dated to approximately 74.5 Ma [54], [55]; and finally *P. tubicen* is found in the Kirtland Formation of northern New Mexico in sediments aged 73.5 Ma [56]. The distributional pattern of *Parasaurolophus* could be easily achieved by a widespread distribution of the genus prior to 76 million years as concurrently observed in Saurolophinae taxa; however, no specimens are currently known from this time in the southern WIB to confirm this speculation.

### Ceratopsid Campanian Biogeography

The subclade Chasmosaurinae has a broader known stratigraphic range (from the late Campanian to the terminal Maastrichtian) than its sister-clade Centrosaurinae [16] with species known from the interval 76–65 Ma. The ancestral area for Chasmosaurinae is posited through DIVA to be the Northern WIB during the Early Campanian (Fig. 3). Subsequently, a dispersal event into the Southern WIB led to a widespread ancestor of the most exclusive clade including *Mojoceratops*. A vicariance event occurring no later than the middle-late Campanian resulted in the northern isolation of the *Mojoceratops* lineage from the ancestor of the most exclusive clade including *Agujaceratops*, in the Southern WIB, which is indicative of two isolated evolutionary centers during the late Campanian [16], [40]. The split of *Kosmoceratops-Vagaceratops* from the species-rich Maastrichtian *Anchiceratops*-*Triceratops* clade is inferred to have been the result of either a vicariance or dispersal event. Contrary to the smaller geographic ranges exhibited by chasmosaurines in the late Campanian, Maastrichtian species were wider ranging (e.g., *Triceratops horridus* [57]).

Centrosaurine ceratopsids have dominantly been recovered from sediments in Alberta and Montana, although a growing body of fossil data from southern Utah is providing information on the earliest Campanian stratigraphic distributions. Sampson and Loewen [16] describe the phylogenetic, geographic, and stratigraphic distribution of centrosaurines in detail; therefore, only information relating to this study will be presented. Centrosaurine taxa that occur in the early Campanian are found throughout the WIB, yet they are not co-occurring, and geographic distributions across large distances cannot be ascertained. However, around 75.5 Ma a new species found in the Kaiparowits Formation is contemporaneous with *Centrosaurus apertus* in Alberta, although they are found in separate clades. This illustrates another example of isolated evolutionary centers at the same time as observed in chasmosaurine ceratopsids and both clades of hadrosaurids. The remainder of the centrosaurine data presented in Sampson and Loewen [16] does not contribute further information to this study.

### Onset of the Laramide Orogeny and Dinosaur Cladogenesis

Several recent studies have used the established connection between phylogenetic relationships and tectonic activity to better understand the relationship between evolutionary patterns, changing geography, and onset of orogenic events [32], [36]–[39]. Our analyses on biogeography and diversification rates in megaherbivorous dinosaurs allow for independent testing of hypotheses regarding the timing and influence of Laramide tectonics.

Our combined analyses suggest that geographic and ecological barriers created from incipient Laramide uplift, in combination with the presence of the Sevier Orogenic Belt and the KWIS, caused initial isolation of northern and southern dinosaurs that ultimately led to the establishment of geographically restricted evolutionary centers. Several predictive tests of speciation via tectonic processes outlined by Badgley [35] can be applied to test this hypothesis: 1) increased levels of endemism should be present in regions affected by tectonic speciation, 2) speciation rates should be greater in topographically complex regions, and 3) correspondence should exist between tectonic activity and cladogenesis.

#### Test 1: Tectonics and endemism

Our analyses demonstrate that hadrosaurid and ceratopsid endemism increases in the earliest stages of the Laramide uplift as defined by geologic evidence. DIVA results (Fig. 3) provide the first rigorous support for the hypothesis that chasmosaurine ceratopsids exhibited widespread geographic distributions throughout the WIB during the early Campanian [40]; additionally, this study is the first to demonstrate that hadrosaurids are also widespread during the early Campanian (Figs. 2 and 4). Subsequently (approximately 75.5 Ma), megaherbivore lineages exhibit more restricted ranges, appearing to have been isolated to either Montana-Alberta (*Prosaurolophus, Chasmosaurus*) or southern Utah (*Gryposaurus monumentensis, Kosmoceratops, Utahceratops* ). Vicariance events during the middle and late Campanian are also supported by ancestral area reconstructions. Restricted endemism terminated during the Maastrichtian when the KWIS retreated allowing taxa to roam eastward and inhabit wider geographic ranges [9] (Figs. 2, 3, 4).

Development of more stringent climatic and ecologic regimes may have played an important role in the isolation of hadrosaurid and ceratopsid faunas in the late Campanian. Examples from the modern record [58]–[60] indicate that ecosystems alter in composition along with subtle variations in elevation incited by orogenic uplift. Although lower global temperature gradients may have mitigated this effect in the Campanian to some degree, changing topography in the WIB would undoubtedly have spurred changes in ecosystem composition. As Late Cretaceous orogenesis commenced weather patterns that were previously dictated by Sevier-induced topography would begin to change because of newly introduced landforms, albeit small at first, which over time would rise to significant heights. Profoundly, changes in elevation would alter air currents and local climate regimes, which would have the compounding effect of changing annual temperatures and rainfall averages. Plant communities have been shown to be sensitive to subtle changes in altitude, rainfall, and humidity [58], [61], [62] and these conditions ultimately dictate local plant composition. McLachlan et al. [63] found that trees migrate at a slower rate than previously appreciated. Therefore, the climatic changes periodically occurring as a result of orogenic uplift and basin segregation may have occurred sufficiently fast enough to outpace plants from crossing these barriers, and thereby altering ecosystems substantially enough to create isolated biomes. This hypothesis is supported by documented palynomorph evidence, which indicates isolated pollen provinces that have long been recognized in Late Cretaceous sediments [64]. Modified plant communities may have acted in combination with potential geographic barriers (such as the Castlegate river/delta system and Wind River Mountains) to spur ecological barriers [65] to herbivorous dinosaurs, preventing gene flow, and creating endemic centers of megaherbivorous dinosaur evolution.

#### Test 2: Late cretaceous diversification rates

SymmeTREE results clearly establish that hadrosaurids and ceratopsids experienced significant variations in diversification rates. If taxonomic diversity reflects net diversification rate, there exists higher diversification for Campanian hadrosaurids and ceratopsids when compared to Maastrichtian rates. Our data show that in a small window of the Campanian when several topographic features (Sevier Orogenic Belt, KWIS, and Laramide structures) coincided, new megaherbivorous dinosaur species appeared at average rates of more than one species per million years, as opposed to the Maastrichtian rates of one species per several million years. If one compares the diversity in hadrosaurids and ceratopsids only in two formations within the northern region of the WIB, there is direct evidence of seven hadrosaurid taxa [15], [66] with additional ghost lineage evidence of two taxa, and direct evidence of at least five ceratopsids [16] found in the 1.5 Myr Dinosaur Park Formation with additional ghost lineage [67] evidence of one taxon, whereas evidence exists of only one hadrosaurid (*Edmontosaurus regalis*) and up to three ceratopsids (*Triceratops horridus, T. prorsus* and possibly *Torosaurus latus*) from the 1.37 Myr Hell Creek Formation [68], [69]. Even more dramatic is the evidence that during the entire 5 million year Maastrichtian stage preserved in the northern Western Interior Basin, there are only four hadrosaurids documented [66], [70] (*Hypacrosaurus altispinus*, *Saurolophus osborni*, *Edmontosaurus annectens*, and *E. regalis*) with no ghost lineage additions, and three to five ceratopsids [16], [68], [71] (*Eotriceratops*, *Nedoceratops*, *Torosaurus latus Triceratops prorsus*, and *T. horridus*), with up to five ghost lineage taxa. These data are not biased on sampling due to nearly 150 years of prospecting in both time periods, and greater outcrop area of the Maastrichtian strata. Additionally, Campione and Evans [70] found a plausible Maastrichtian drop in megaherbivore diversity based on morphological disparity. Therefore, net diversification rates slowed in correspondence with an increase in habitable land, as predicted.

#### Test 3: Tectonic correspondence

The phylogenetic and geographic range data for megaherbivorous dinosaurs support the hypothesis that the thrust of clade diversification is contemporaneous with the establishment of Laramide uplift between southern Alberta and southern Utah approximately 75 Ma. More specifically, the Wind River Mountains of southern Wyoming started cooling between 85–75 Ma [23], and the Big Horn Mountains began cooling as early as 70 Ma [24]. The Rock Springs and Douglas Creek uplift initiated simultaneously in the Campanian [72]. Uplift of the Colorado Plateau reached two peaks of maximum ascension rate at approximately 80 Ma and 70 Ma [73]. Several drainage basins in southern Utah that date to approximately 74.5 Ma show increased subsidence rates and changes to flow direction hypothesized to be part of the Laramide uplift [28], [29]. The Castlegate river delta system deposited as a result of uplift of the Charleston-Nebo salient in the early Campanian [74] (∼77 Ma). Together, these data firmly establish a correspondence between the main thrust of dinosaur cladogenesis as documented by fossil evidence and establishment of incipient Laramide tectonic structures as evidenced by the geologic record. However, our phylogenetic analyses time the initial divergence of *Chasmosaurus* and *Pentaceratops* clade ceratopsids and *Gryposaurus* and *Prosaurolophus* clade hadrosaurids at approximately 78.5 Ma, suggesting that topographical surficial deformation caused by the Laramide orogeny may have been present in the early Campanian. This date precedes estimates based on substantial sedimentological evidence and, if plausible, provides the earliest evidence for the Laramide orogeny north of Arizona/New Mexico [75]–[78].

### Conclusion

We provide quantitative evidence that rapid cladogenesis of Campanian megaherbivores occurred coincidentally with incipient confluence of the Sevier Orogenic Belt, KWIS, and Laramide tectonics, and that once the Laramide uplift was substantial enough to drive regression of the KWIS during the Maastrichtian, net diversification rates followed suit. Phylogenetic divergence estimates of fossil clades offer a new lower boundary on Laramide surficial deformation that precedes estimates based on sedimentological data alone.

Application of these results to other dinosaur groups contemporaneously living in Laramidia is an interesting prospect. The major hurdle to such comparative studies is insufficient fossil records of other clades, although based on limited data theropods may exhibit similar trends. Different species of tyrannosaurid and troodontid are known to live within the northern and southern WIB approximately 75.5 Ma [79], [80]. Once established, comparison of the trends observed within ceratopsids and hadrosaurids in response to the unique geologic and topographic conditions spotlighted in this study to those trends observed in other dinosaur clades will allow insights into the tempo and modes of evolutionary change among the dominant terrestrial vertebrates of the Cretaceous.

## Supporting Information

SymmeTREE Information S1 **Data output files from SymmeTREE analysis of ceratopsid and hadrosaurid phylogenies.**
(XLSX)
