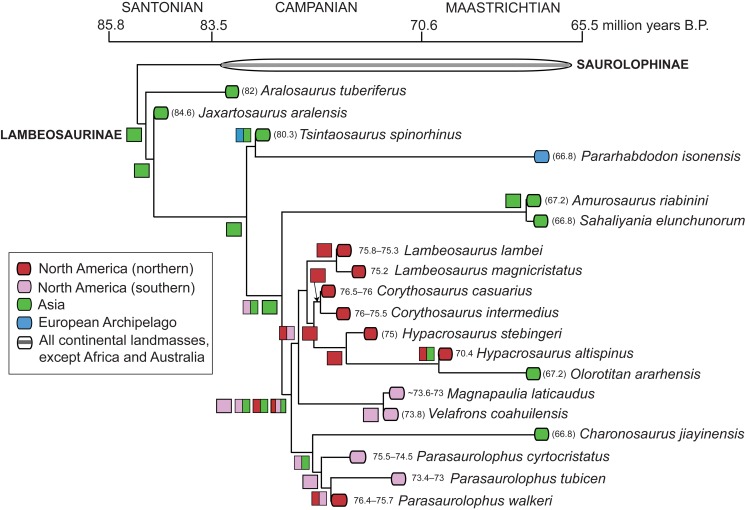# Correction: Mountain Building Triggered Late Cretaceous North American Megaherbivore Dinosaur Radiation

**DOI:** 10.1371/annotation/3807b04a-974d-4725-9430-baf742117aa4

**Published:** 2012-10-17

**Authors:** Terry A. Gates, Albert Prieto-Márquez, Lindsay E. Zanno

Figure 5 is incorrect. 

The correct figure can be found here: 

**Figure:**